# Plasmonic- and dielectric-based structural coloring: from fundamentals to practical applications

**DOI:** 10.1186/s40580-017-0133-y

**Published:** 2018-01-10

**Authors:** Taejun Lee, Jaehyuck Jang, Heonyeong Jeong, Junsuk Rho

**Affiliations:** 1 Department of Mechanical Engineering, Pohang University of Science and Technology (POSTECH), Pohang, 37673 Republic of Korea; 2 Department of Chemical Engineering, Pohang University of Science and Technology (POSTECH), Pohang, 37673 Republic of Korea

**Keywords:** Structural color printing, Color filters, Plasmonic color filters, Plasmonics, Dielectric color filters, Metasurfaces, Metamaterials, Sub-wavelength, Nanophotonics, Tunable color filters, Large scale color filters, Up-scale color filters

## Abstract

Structural coloring is production of color by surfaces that have microstructure fine enough to interfere with visible light; this phenomenon provides a novel paradigm for color printing. Plasmonic color is an emergent property of the interaction between light and metallic surfaces. This phenomenon can surpass the diffraction limit and achieve near unlimited lifetime. We categorize plasmonic color filters according to their designs (hole, rod, metal–insulator–metal, grating), and also describe structures supported by Mie resonance. We discuss the principles, and the merits and demerits of each color filter. We also discuss a new concept of color filters with tunability and reconfigurability, which enable printing of structural color to yield dynamic coloring at will. Approaches for dynamic coloring are classified as liquid crystal, chemical transition and mechanical deformation. At the end of review, we highlight a scale-up of fabrication methods, including nanoimprinting, self-assembly and laser-induced process that may enable real-world application of structural coloring.

## Introduction

Color production mechanism are mainly classified by two types: pigmentary or structural coloring. Structural colors, in particular, are caused by microscopic structures that are tiny enough to interfere with visible light. In nature, structural coloring occurs among birds and insects [1–6]. This method of generating colors has inspired the field of structural color printing. Many artificial and biomimetic colors from nature have been reproduced [7, 8] and applied to photonic crystal research [9–11]. However, the diffraction limit of light presents a challenge to further development of structural color printing. Recent developments of techniques to fabricate metal-based structures have shown a way to overcome the diffraction and to approach nano-size resolution.

Surface plasmon resonance (SPR) by the electric field along a metallic surface can confine optical excitation to far below the diffraction limit [12, 13]. Furthermore, SPRs can be used to manipulate the polarization, phase and intensity of light [14–18, 19, 20, 21]. These characteristics offer a capability to generate structural colors from SPRs on plasmonic structures. Plasmonic color filters (PCFs) can have sub-wavelength unit cells and surpass the diffraction limit of light due to SPR from plasmonic structures [22, 23, 24]. Hole-array PCFs achieve high transmittance by exploiting extraordinary optical transmission (EOT) caused by SPRs [15, 25–27].

The metallic layer in PCFs absorbs visible light; this phenomenon can reduce their efficiency. Use of dielectric-based color filters (DCFs) supported by Mie resonance has been suggested as a method to circumvent this problem [28–30]. DCFs have relatively low loss, so they can control bandwidth adaptively. Additionally, owing to optically-generated electric and magnetic resonances and low cost, DCFs have substantial potential to complement or even replace pigments and PCFs [31].

Structural coloring has limitations such as static color, limited-scale fabrication and low throughput. Structural filters produce colors that stay mostly static, so the search for a method to tune them is currently an important research topic. Structural color filters that can be tuned by adjusting external factors can manipulate colors by controlling factors such as polarization angle of incident light, alignment of liquid crystals (LCs) [32–35], mechanical strain [36–39] and chemical state [40–43]. In this review, we summarize research on plasmonic color filtering, with a brief explanation of its working principle. We also introduce recent developments in tunable color filtering and large-scale color filtering, which may lead to real-world application.

## Plasmonic color filters

Color filters selectively reflect or transmit light of a target wavelength. A unit (pixel) in an array transmits dominantly one color (e.g., red, green, or blue). Each pixel delivers different color information, and a combination of colored pixels can produce a specific image. A structural color filter has a nano-scale structure that interacts with incident light to reflect or transmit light of a specific wavelength. PCFs that exploit plasmonic resonance are promising candidates to replace conventional pigment- or dye-based color filters. We will review research on PCFs sorted by structure.

### Nanohole

Transmission of light through a subwavelength aperture in regularly patterned opaque metal film is enhanced at resonant wavelengths. This phenomenon is called EOT, and is one of the most important recent discoveries in optics [15]. The effect is associated with a coupling between excitation of surface plasmon (SP) and incident light in a metallic surface [44, 45]. The interaction can be manipulated by tuning geometric parameters such as periodicity, size and shape of apertures. This observation has triggered a wide variety of related research [46–51].

**Fig. 1:**
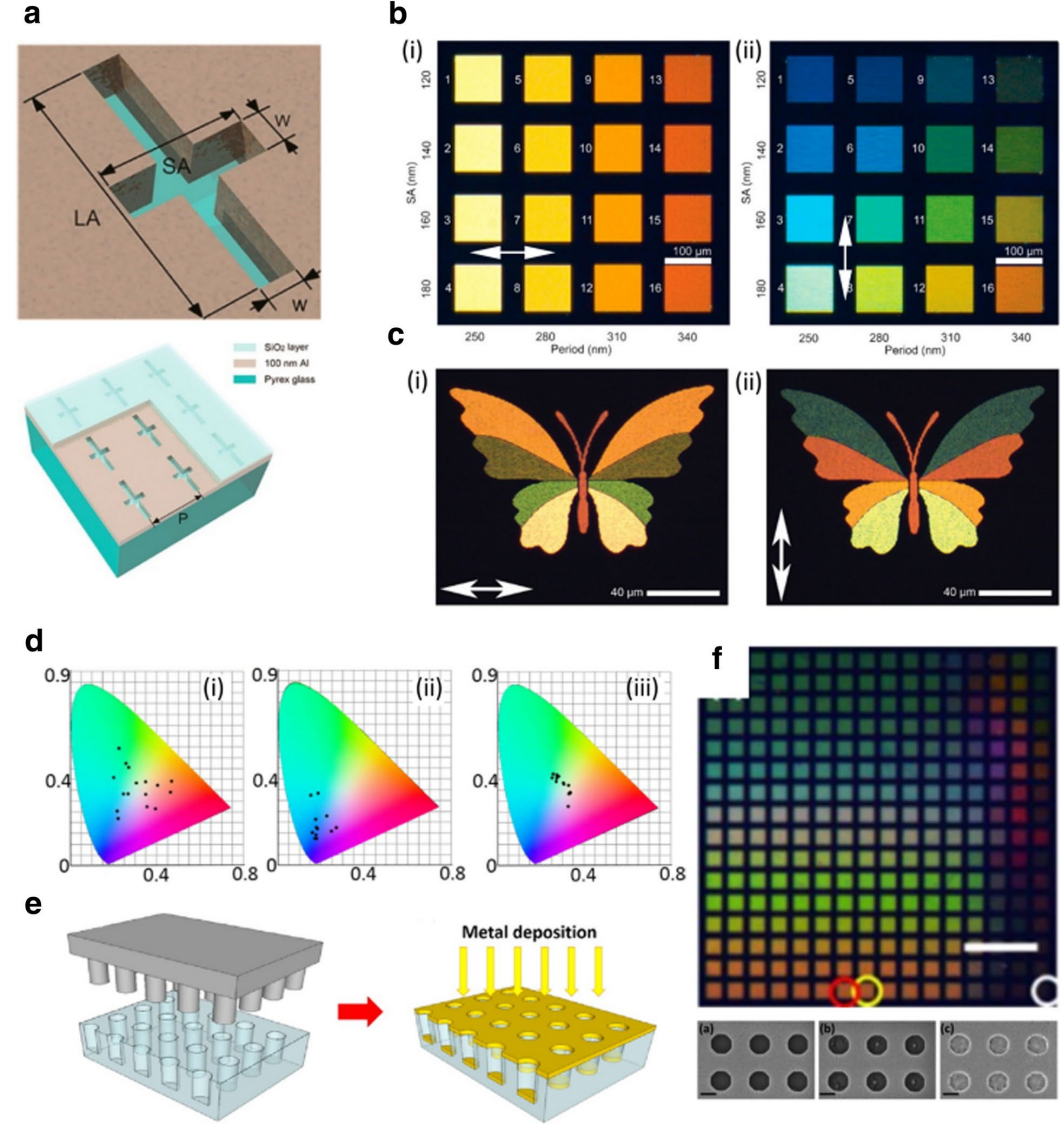
**a** Schematics of cross-shaped PCFs (*SA* short arm, *LA* long arm. The average dimensions of aperture are *w* = 30 ± 2 nm, *P* = 340 nm, SA = 120 ± 5 nm and LA = 203 ± 3 nm. **b** Color palette with variation in polarization. (i) Colors transmitted through the LA resulting from polarization along the SA. (ii) Colors transmitted through the SA resulting from polarization along the LA. **c** Micrographs of butterfly with wing color variations, when polarized along SA (i) and LA (ii) [52]. **d** Color distributions simulated in CIE 1931 chromaticity diagram with (i) Ag (ii) Al and (iii) Au. **e** Two-step fabrication composed of nanoimprint and metal deposition. **f** (top) Transmissive microscopic color palette using Ag. (bottom) Three scanning electron microscope (SEM) images of nanohole pixels with 500-nm periodicity and exposure to e-beam dose of 900 (left), 720 (middle) and 500 (right) μC/cm^2^, corresponding to red, yellow and white circles of top image [53]

In PCFs with asymmetric cross-shaped apertures, one nanoaperture can generate two colors (Fig. 1a). PCFs in aluminium (Al) film can have polarization-adjustable properties that result in selectivity between two transmitted colors [52]. By manipulating geometric parameters such as long arm (LA), short arm (SA) and periodicity of nanocavity apertures, various colors are obtained. Exchanging the polarization directions of the LA and SA result in dual color variations. No color changes in SA-variable palettes appear at a same period when light is transmitted through the LA and polarized along the SA. In contrast, the color changes gradually when light is transmitted through the SA and polarized along the LA (Fig. 1b). Therefore, by controlling polarization direction of incident light an operator can selectively obtain one of two colors from the plasmonic device.

Nanohole-shaped PCFs based on silver (Ag) instead of Al or gold (Au) can produce color-enhanced transmissive structural colors [53]. The Ag color diagram has a wide distribution of displayed colors, whereas the Al and Au diagrams focus on certain colors (Fig. 1d). Authors also present three pixels which are resists that were fully, partially and barely exposed to electron beam lithography (EBL) (Fig. 1f, red, yellow and white circles). Although the holes are barely exposed, they show no variation of either color or shape compared to fully-exposed pixels. The fabrication method is a simple two-step process of nanoimprinting and depositing the metal (Fig. 1e). Because this fabrication is simple and compatible with large-scale fabrication, commercialization of these devices as color filters is expected.

### Metal–insulator–metal

Metal–insulator–metal (MIM) nanoresonators with an insulator sandwiched between two metallic materials can also act as color filters. The key principle of MIM is Fabry–Pérot interferometry, in which interference of light within a resonator selectively filters out light of a certain wavelength. The design of the MIM has evolved from films to 2D metasurfaces with unit structures such as gratings or posts [54–64]. To improve efficiencies or color purity, researchers have changed basic materials [65–67].

**Fig. 2:**
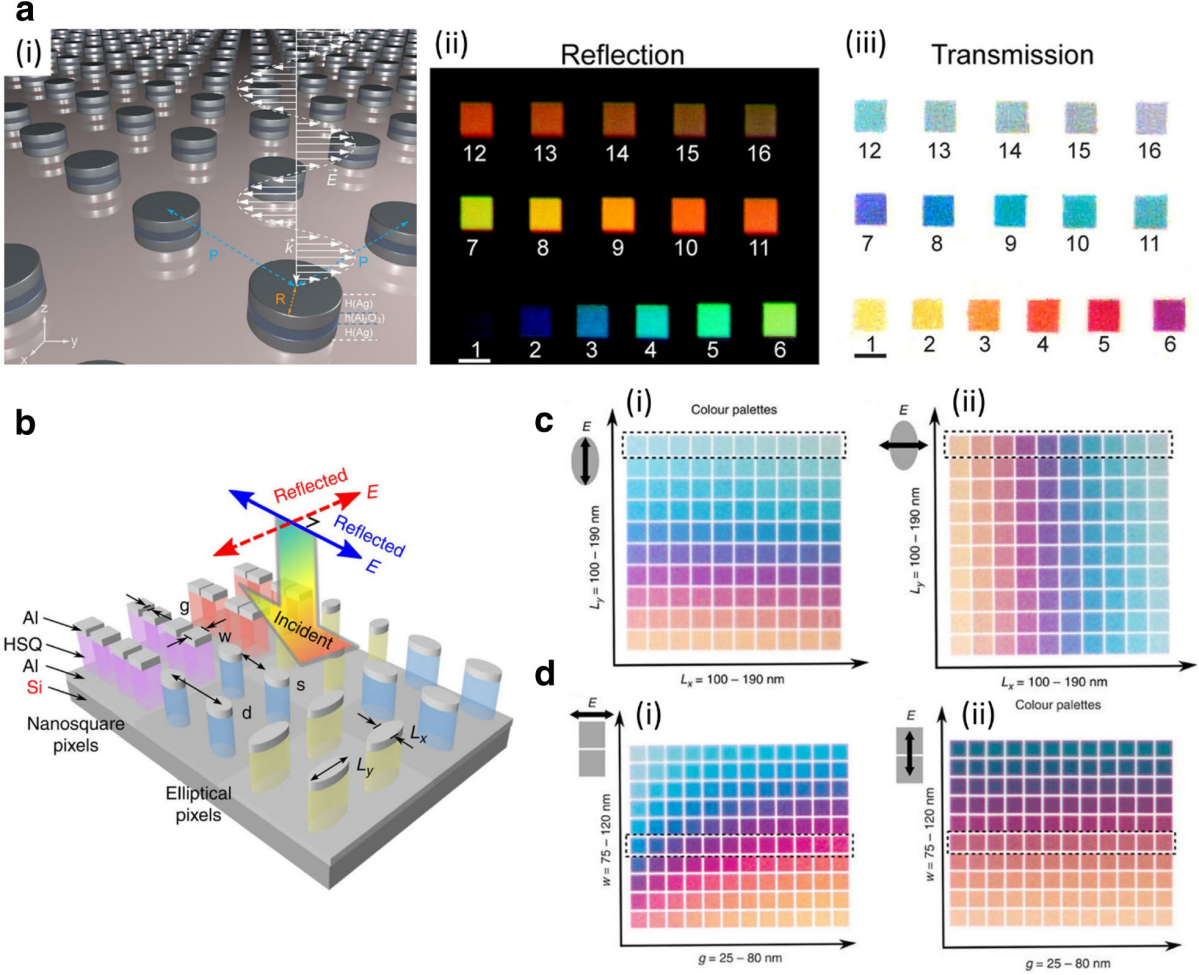
**a** (i) Schematic design of tandem nanodisk array. Measured (ii) reflective and (iii) transmissive color images corresponding to (i) with radii 35 ≤ *R* ≤ 110 nm and periods 250 ≤ *P* ≤ 450 nm. Scale bar: 40 µm. **b** Schematics of a plasmonic stereoscopic printing device with dependence on polarization. **c** Optical color palettes for elliptical pixels varied by Lx and Ly, polarized along (i) *y* and (ii) *x* direction. **d** Optical color palettes for coupled nanosquare pixels at combinations of w and g, polarized along (i) *x* and (ii) *y* direction

A research based on Ag-alumina (Al_2_O_3_)–Ag exploiting tandem nanodisks demonstrates a wide range of color generation by adjusting geometric parameters such as periodicity and radius of structures (Fig. 2a) [68]. Previous works on plasmonic color printing have mostly concentrated on hue instead of color brightness and saturation. These methods suffer from relatively broad full width at half-maximum, shallow peaks, and dips in spectra. A device based on Ag–Al_2_O_3_–Ag consists of an array of tandem nanodisks to solve these limitations, and achieved bright and saturated colors with red, green and blue in reflective mode, and cyan, magenta and yellow in transmissive mode (Fig. 2ai, ii). These complementary near-full colors are attributed to a combination of Wood’s anomaly and an in-phase electric dipole. This device is relatively easy to fabricate because it uses comparatively large nanodisks.

Plasmonic stereoscopic printing has been demonstrated [69]. The device can produce a great variety of colors in reflective mode by using polarization-dependent tunable color pixels in pairs of arrays of squares and of ellipses (Fig. 2b). Dual color information can be encoded by two polarization directions in the same pixel (Fig. 2c, d), so this printing technology may have applications as high-density optical data storage, high-resolution 3D display, holograms and anti-counterfeiting measures.

### Nanorod

**Fig. 3:**
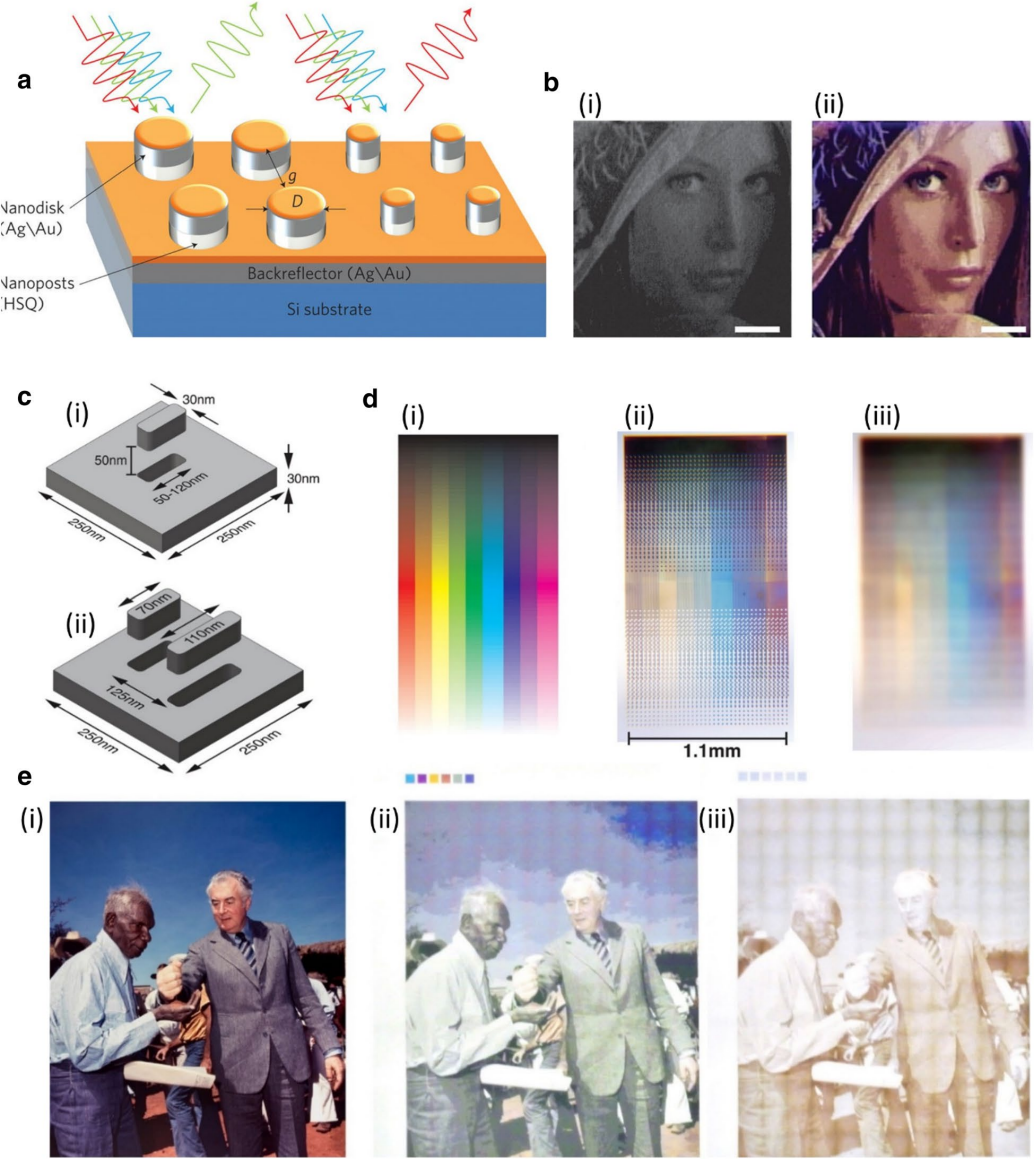
**a** Schematic illustration of nanorod-based color printing device. Wavelength of reflected light is changed by varying diameter *D* and separations *g* of nanodisks. **b** Microscopic images of Lena (i) before and (ii) after deposition of a metallic capping layer. **c** Schematics of floating dipoles of plasmonic pixels producing (i) subtractive colors and (ii) black. **d** (i) Test pattern to observe variations of brightness and saturation via control of plasmonic pixels. (ii) ×5 magnified image of manufactured pattern of test structures, showing arrangement of the plasmonic pixels. (iii) Defocused magnified image of test pattern indicating gradual variations of brightness and saturation. **e** (i) Original photograph of Vincent Lingiari and Gough Whilam in 1975 by Mervyn Bishop. Reproduced images polarized along (ii) *x* axis and (iii) *y* axis

Color imaging devices based on nanorod arrays can be made to demonstrate a color spectrum by manipulating their resonances [70–76]. The generated colors are mainly attributed to an interaction between plasmon resonance that is related to peaks in spectra, and Fano resonance that is related to dips in them. Although the influence of the Fano resonance is not critical, it helps to narrow the spectral peaks to sharpen and enrich the colors. Near-full-color printing has been achieved using Ag and Au nanodisks on a backreflector that encodes the color information (Fig. 3a) [77]. Each color pixel consists of four nanodisks; the results are achieved by varying the diameter of disks and the gap between them, but are not affected by their periodicity. Use of a metallic capping layer can also intensify the color (Fig. 3b). This approach is compatible with nanoimprint lithography, and may therefore be amenable to scale-up and high-throughput fabrication.

A novel nanorod-shaped plasmonic pixel can produce optical resonances over the entire visible spectrum [78]. The floating plasmonic pixel acts as a plasmonic nearly-perfect absorber with narrow bandwidth, and thereby produces immensely-saturated subtractive colors (Fig. 3ci). The yellow, magenta and cyan of subtractive colors are generated at dipole lengths of 70, 90 and 120 nm, respectively. Experimental results agree well with simulation, except in saturation of the yellow. The discrepancy may result from fabrication imprecision, which broadens a shape of the resonance, but can be overcome by manipulating evaporation parameters. Black is generated by a two-connected floating dipole that acts as a near-perfect absorber, so broad absorption appears in the visible spectrum (Fig. 3c) [30]. This approach can also adjust the color response by tuning geometric parameters such as nanoantenna length and gap between antenna and film.

### Grating

Development of grating-based 1D PCFs has achieved > 70% average efficiencies of either transmission or reflection [49, 79–89]. Due to structural periodicity, these devices usually exhibit angle-sensitivity and the momentum-matching constraint of surface plasmon polaritons (SPPs). These problems have been resolved simultaneously using randomly-arrayed nanostructures [90].

**Fig. 4:**
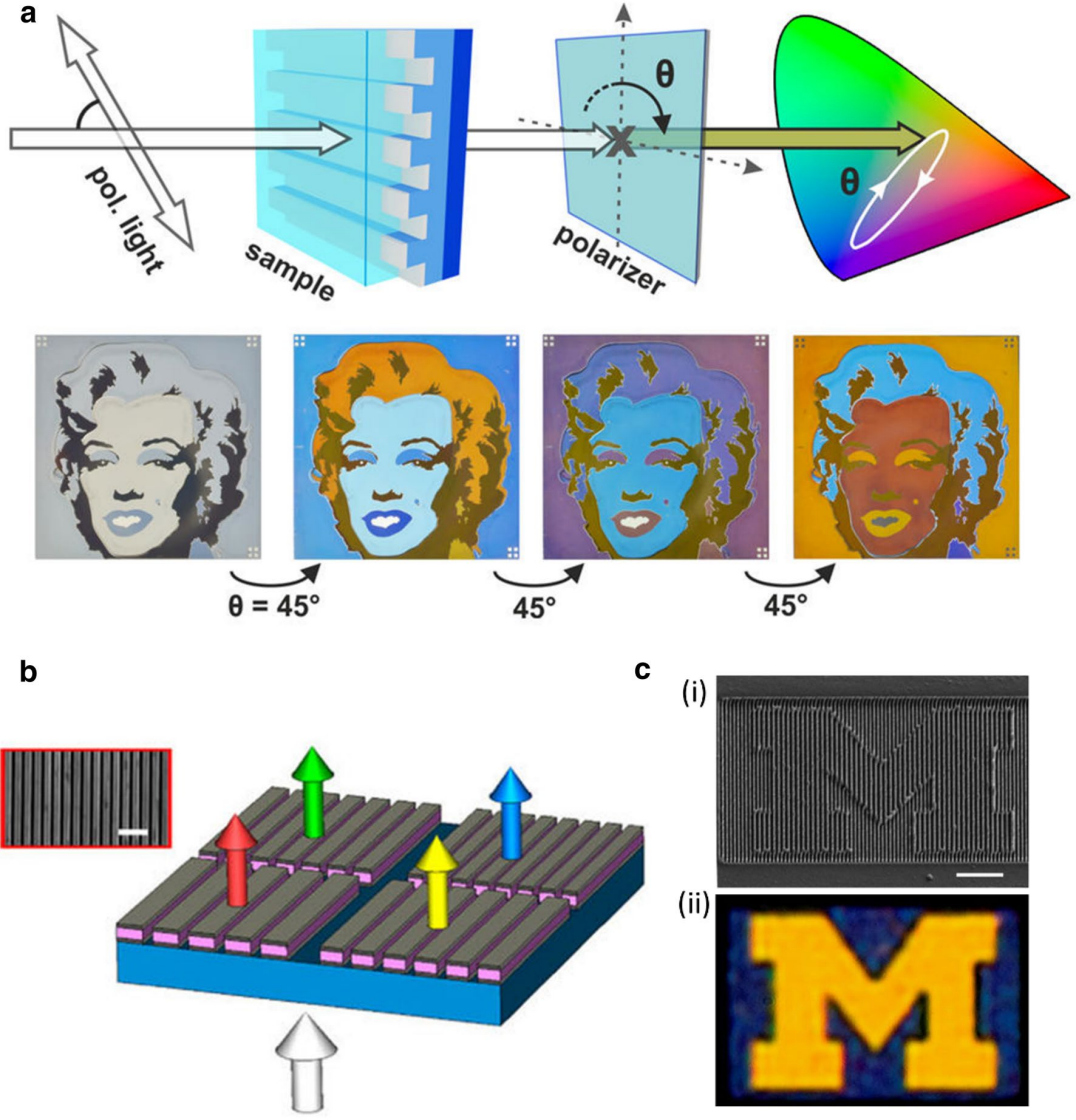
**a** Schematic of silver-based grating color filter using plasmonic phase retarder. Four Marilyn Monroe images illustrate effects of increasing the angle of the incident polarization [91]. **b** Schematic design of a MIM grating coloring device. Incident white light is filtered into resonant wavelengths (colors). Inset: SEM image of a design; scale bar: 1 μm. **c** (i) SEM image of letter ‘M’ with 220-nm background period and 310-nm period of ‘M’. (ii) Optical micrograph of (i) illuminated using white light [62]

Polarization-dependence can also appear in grating-based color-printing devices. A silver-based grating color filter that uses a plasmonic phase retarder causes dependence on incident polarization by simple rotation of a polarizer [91]. By changing the different polarizer settings, the same input image can be given very different color properties (Fig. 4a). The 45° and 135° polarization angles generate strongly-contrasting colors of purple, dark blue, orange and yellow. Photomask used to make the images is divided into five areas according to the grating thickness of the Ag layer. The analyzing polarizer has two inverse regions to improve optical appearance. Polarization-sensitive color printing technology has potential applications in security [92, 93], sensing [94], polarizing detectors [76] and magneto-optics [95].

A combined design of MIM and grating can efficiently separate white light into a certain wavelength with distinct color (Fig. 4b) [62]. The MIM-grating color filter consists of Al–ZnSe–Al resonators. Diffraction at the bottom Al grating layer helps couple incident light to plasmon waveguide modes; scattering at the top Al grating layer reconverts the detained plasmon to a propagating wave. The ZnSe layer with 100-nm thickness encourages a coupling of SP in top and bottom, so that the nanoresonators effectively actualize a conversion of photon–plasmon–photon at the resonant wavelengths. This MIM grating design has many advantages of compactness, effective transmission and narrow passband. The dependence on polarization eliminates the need for an individual polarizer layer, so these MIM/grating devices may have applications in liquid crystal displays [96].

## All-dielectric structure supported by Mie resonance

Devices that use metallic nanostructures to induce structural colors can manipulate light absorption and scattering beyond the optical diffraction limit. These devices have advantages of compactness, high resolution, robustness, and compatibility with integration in various devices. DCFs have been studied to seek capabilities that complement limitations of metal, such as high loss that leads to peak broadening. Such devices exploit Mie resonance based on Mie scattering that depends on both the geometry and size of particles. In principle, dielectric nanoparticles (NPs) with high refractive index *n* can affect the results, with both the electric dipole and magnetic dipole having comparable contributions, whereas PCFs mainly control the electric dipole. The resonant magnetic response is caused by coupling of incident light toward the circular displacement current of the electric field as a result of retardation of phase and field penetration. This series of processes arises when the wavelength *λ* of the light is approximately the diameter 2*R* of inner particles (2*R* ≈ *λ/n*). This mechanism may also provide an opportunity to design DCFs that use Mie resonances to exploit higher-order multipoles [31].

Optically-generated resonant magnetic responses in dielectric NPs have been observed in particles with different geometries such as ring [97], spheroid [98, 99], disk and cylinder [100] and sphere [101]. This diversity of optically-active shapes provides an opportunity to create diverse all-dielectric nanostructures by varying the geometric parameters of NPs to adjust both magnetic and electric resonances. Before choosing design of structure, its material of structure must be selected, because it effects the optical characteristics of the device such as transmission, reflection, optical loss and efficiency.

Many types of dielectric materials are complementarily available; each has advantages and disadvantages. Several materials cannot be used alone because of high optical losses. Hence, these materials are sometimes used in chemical or mechanical mixtures in which each has a property that compensates for the weakness of another. So far, silicon (Si) has been used most commonly, but many researchers have attempted to find optimal materials suitable for devices that require particular optical properties.

### Pure Si

Si have been frequently used in printing technology because of low cost, reliability and compatibility with optoelectronic devices. Si has a high *n*, and can therefore manipulate light subwavelength scale [102, 103]. Importantly, Si particles with subwavelength size exhibit strong, optically-induced magnetic and electric Mie resonances at visible wavelengths. By exploiting this optical property, Si nanowires can be used as color filters to convert absorbed light to photocurrent [104]. Si can also efficiently tailor the symmetry of light emission and enhance magnetic radiative decay [105–109]. Many studies have used pure Si in a variety of geometries such as nanopillars [110] and crosses [111]. The studies mainly accomplished their goals of obtaining high-quality resonances in the entire visible range, which yield a great color gamut of high-purity colors. In devices with cross-shaped Si nanoresonators, the high-quality Mie resonances provide good confinement of energy to the structure [111].

**Fig. 5:**
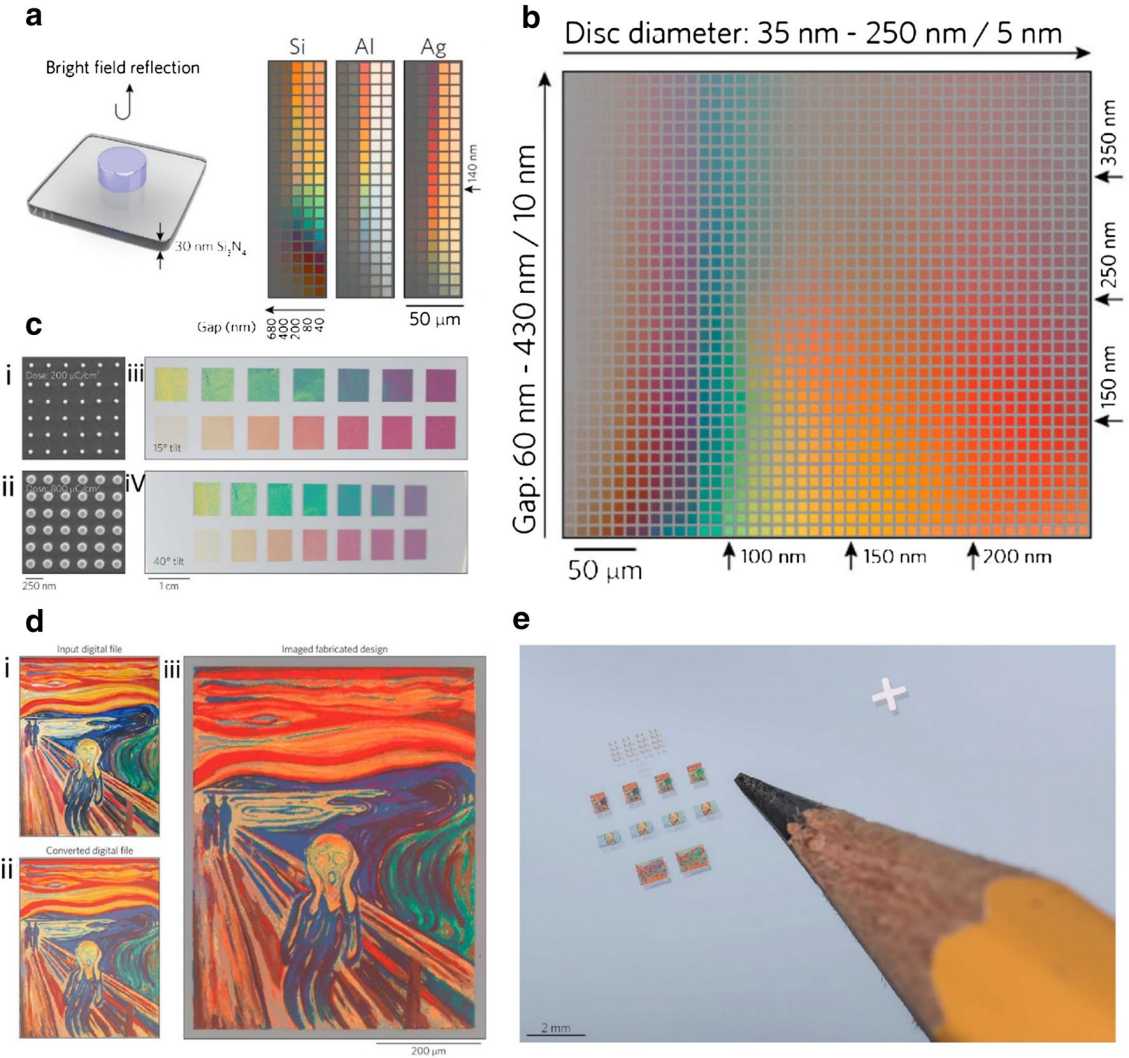
**a** Schematic configuration of nanodisk fabricated on Si_3_N_4_, with optical micrographs of Si, Al and Ag. **b** Optical micrographs of a color filter using Si-based nanodisks (diameters 35–250 nm in increments of 5 nm; gaps between nanodisks are 60–430 nm in increments of 10 nm). **c** SEM images of dot array with a pitch of 250 nm, and dose of (i) 200 µC/cm^2^ and (ii) 800 µC/cm^2^. Square color images (7.5 × 7.5 mm) tilted at (iii) 15° and (iv) 40°. **d** (i) A original painting (The scream by Edvard Munch); (ii) the painting after discretization of the colors generated in (i) of **b**. (iii) Optical micrograph reproduced by the color filter based on Si nanodisk (545 × 700 µm). **e** Photograph of reproduced paintings on a quartz wafer with a pencil for scale [112]

Research using pure Si nanodisks obtained colors and spectra that are distinct from those obtained using metal (Al, Ag) nanodisks [112] (Fig. 5a). Si provided monotonous hue variations, whereas metals exhibit rapid color changes. These differences occur because peaks are clearer in Si spectra than in Al and Ag. Colors and their intensity produced by the DCFs can be adjusted by tuning the diameter and gap of nanodisks (Fig. 5b). This color exhibits little angle dependence (Fig. 5c). A reproduction of a painting replicated the original well (Fig. 5d).

### Enhanced Si and others

**Fig. 6:**
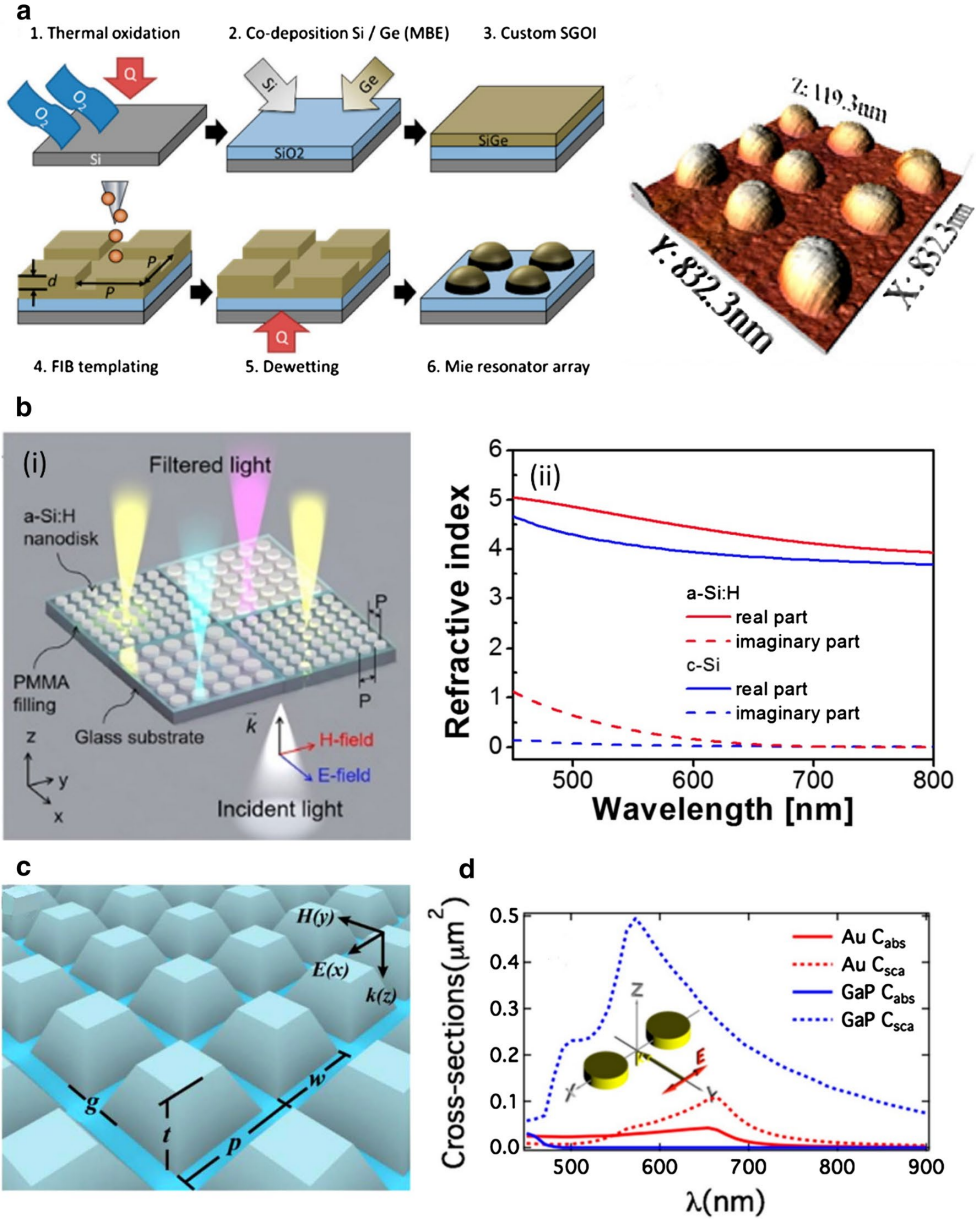
**a** Schematic fabrication steps and AFM image of a dielectric nanoresonator [113]. **b** (i) Schematic design of a structural color filters exploiting amorphous Si:H nanodisks with different diameters *D* and period *P*. (ii) Refractive indices of the amorphous Si:H and crystalline Si:H [28]. **c** Tilted image of a TiO_2_-based metasurfaces [125]. **d** Cross sections of absorption and scattering in case of Au dimer and GaP dimer with disk shape. The disk radii are 50 nm (Au) and 100 nm (GaP) and heights are 37.5 nm (Au) and 75 nm (GaP) [126]

To enhance the optical properties of Si, many researchers have attempted to find improved materials and designs. Germanium (Ge) has been evaluated as a substitute for Si (Fig. 6a) [113]. Ge does not have high-quality resonance due to high absorption in the visible range, and therefore cannot be used alone as a color filter. A material composed of Si (0.8) and Ge (0.2) achieved higher refractive index and lower absorption coefficient than pure Si in most of the visible region. The ratio of Ge must be minimized to avoid high absorption.

Hydrogenated amorphous Si (a-Si:H) has been evaluated as the material in all-dielectric color filters (Fig. 6b) [28]. Many of existing all-dielectric structural filters consist of crystalline Si (c-Si) [10, 114–120], but they have low transmission and a challenge to grow high-quality c-Si on foreign substrates. Compared to c-Si, a-Si:H has advantages of low cost, compatibility with complementary metal–oxide–semiconductor process, and efficient growth on foreign substrates at low temperature to achieve high refractive index. The structural filter based on a-Si:H had a higher refractive index than c-Si (Fig. 6bii). Although this design has some losses at short visible wavelengths, a-Si:H has superior optical properties, low cost, and simple fabrication, and therefore may be an alternative to other color filters.

Various materials such as TiO_2_ and GaP have been evaluated as alternatives to PCFs and other structural color filters [38, 121–124]. TiO_2_ is a reasonable candidate; a recent report (Fig. 6c) obtained a suitable *n* ~ 2.54 at 400 nm, with near-zero extinction coefficient, which means remarkably low loss in the visible range [125]. GaP has also merits in designing all-dielectric metamaterials. The scattering cross section of GaP is ~ 0.5 whereas it is ~ 0.1 in Au disks. The GaP absorption cross section is nearly zero from 500 nm on (Fig. 6d) [126]; the goal of this study was to discover a material that does not suffer from the visible-spectrum losses of PCFs through the comparison with metal (Au). Results may provide a good alternative to metals that exceeds their far-field and near-field emission efficiency.

## Tunability and dynamic modulation of colors

Dynamic color printing is essential for practical applications such as dynamic displays, cryptography and camouflage. Research into tunability of structural coloring is increasing, due a to desire for advanced and innovative functionalities of metasurfaces. Most previous designs could only generate one static color with fixed geometry, but color filtering with tunable function would have a diversity of applications. In this section, we introduce various type of tunable color filters (TCFs) that have studied recently. Methods used include applications of LCs [32–35, 127–129], of chemical transitions [130–133] and of mechanical transformations [36–39].

### Liquid crystals

**Fig. 7:**
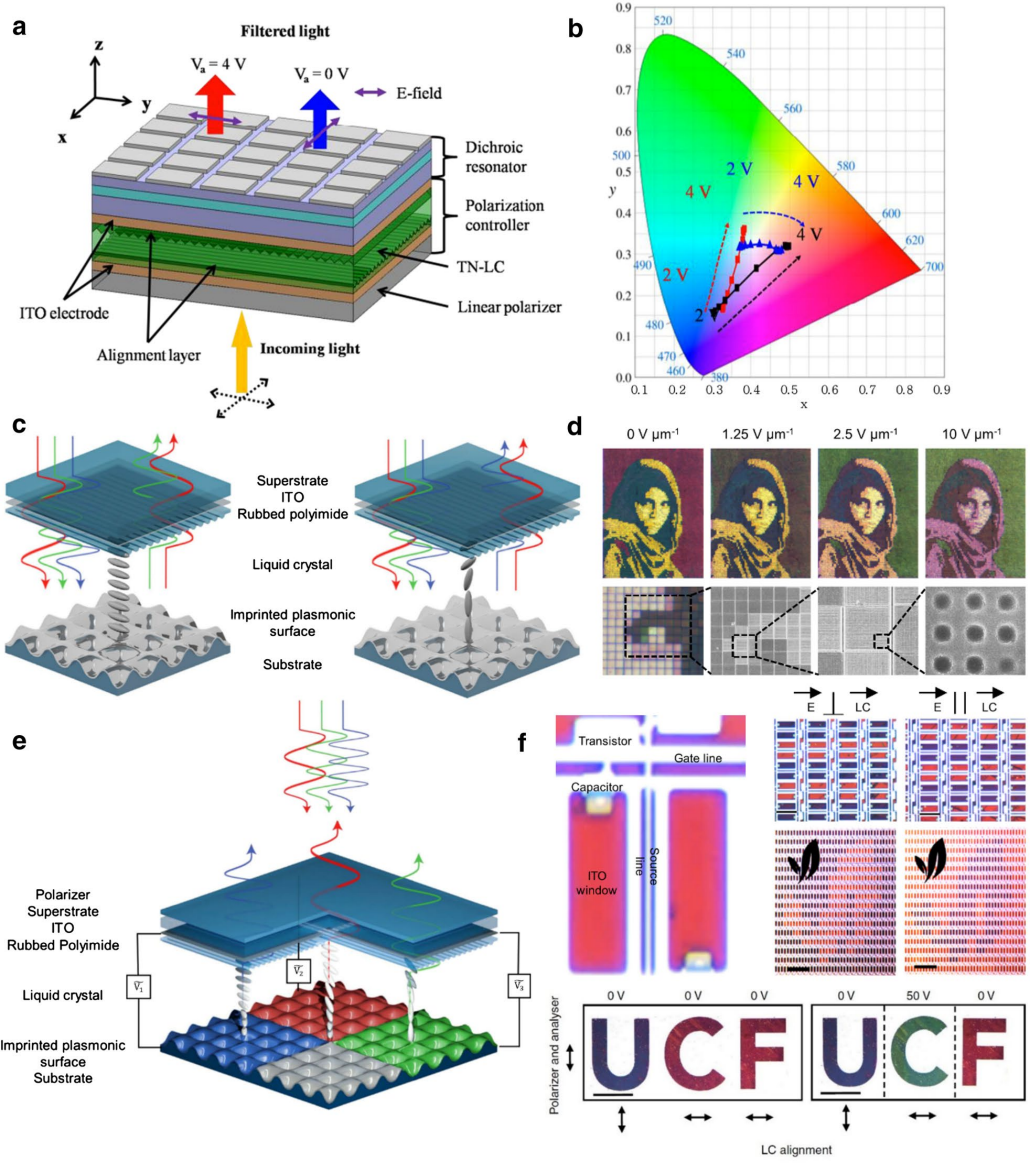
**a** Cell of tunable color filter with LC polarization rotator. **b** Color gamut of proposed dichroic resonator in CIE 1964 color coordinates. Red, blue, black lines represent the BG, GR, and BR sample respectively [127]. **c** Schematic of designed LC-plasmonic system. **d** Afghan Girl image as function of applied electric field. (SEM images below show pixels of imprinted plasmonic surface [128]). **e** Overall design of proposed LC plasmonic system. **f** (Left top) Image taken by a ×10 objective of imprinted plasmonic surface combined with conventional a thin-film-transistor (TFT). (Right top) random image taken by a ×4 objective from integrated device. Scale bars: 0.72 mm. (Bottom) ‘UCF’ patterned device as function of applied voltage, LC alignment and polarizer and analyzer. Scale bars 1.57 mm [129]

One method of achieving tunability is to exploit the anisotropy and rearrangement of LCs under applied voltage; this approach achieved rotating polarization of incident light at will. An electrically-tunable color filter with a LC polarization rotator has been achieved [127] by exploiting guided mode resonance with waveguide layers, and diffracted wave by an asymmetric Al pattern to govern resonance in proposed resonators. The design used Al nanoresonators that have an asymmetric gap distance between the resonators along *x* and *y* directions, and therefore respond differently to *x*-polarized and *y*-polarized light (Fig. 7a). With no voltage applied to a unit cell, polarization of incident light is rotated to 90°, but when applied voltage exceeds a threshold *V*_a_, polarization is maintained at 0°. At voltage < *V*_a_, *x* and *y* polarizations coexist. Transmission spectra by polarization control with voltage manipulation is expressed as *T*_*θ*_ = *T*_0_(*λ*) cos^2^*θ* + *T*_90_(*λ*) sin^2^*θ* where *T*_0_ and *T*_90_ are the transmissions of *x* and *y* polarization, and θ is polarization angle. Consequently, the proposed visible dichroic resonator can be used to tune expressed color in a narrow range of CIE 1964 color coordinates (Fig. 7b).

Tunable color generation can be achieved using an imprinted structure in contacted with LC [128]. This method achieves color tunability by dynamic refractive index tuning by topological reorientation of LC. A shallow imprinted Al layer is surrounded by a high-birefringence LC (Fig. 7c). As unpolarized white light passes through the LC layer, the light couples to plasmonic modes at the imprinted metal surface. The orientation of the LC determines the spectral location of SPR, because SPR Modes depend on the dielectric constant of the surroundings. The LC’s high birefringence causes a large plasmonic shift that leads to high range of color tunability. With no applied electric field, the LC aligns parallel to the Al surface. When an electric field is applied, the LC near the imprinted surface assumes the orientation state that minimizes its internal energy. As the voltage of the electrical field is increased, the LCs keep changing their orientation until they are all normal to the surface. This method achieves higher dpi than a conventional display, and has millisecond-scale response times (Fig. 7d). This research demonstrates the benefits of the LC-plasmonic system, and suggests a method to achieve TCFs.

This tunable device composed of imprinted structure with LC has been shown to be compatible with thin-film-transistor (TFT) technology [129]. The imprinted plasmonic surface was surrounded by a highly birefringent LC and stacked, followed by rubbed amide, ITO, and a supersubstrate layer (Fig. 7e), and the stacked layers were integrated with a TFT array (Fig. 7f). The integrated device is connected to computer so that individual pixels are manipulated via images that the monitor displays. The authors also generated full images and a video of text editing (Fig. 7f, bottom). Although prototype had demerits including image degradation by white reflection from TFT metal lines, and inability to source high voltage, this LC-plasmonic devices shows the possibility of replacing conventional displays.

### Chemical transition

**Fig. 8:**
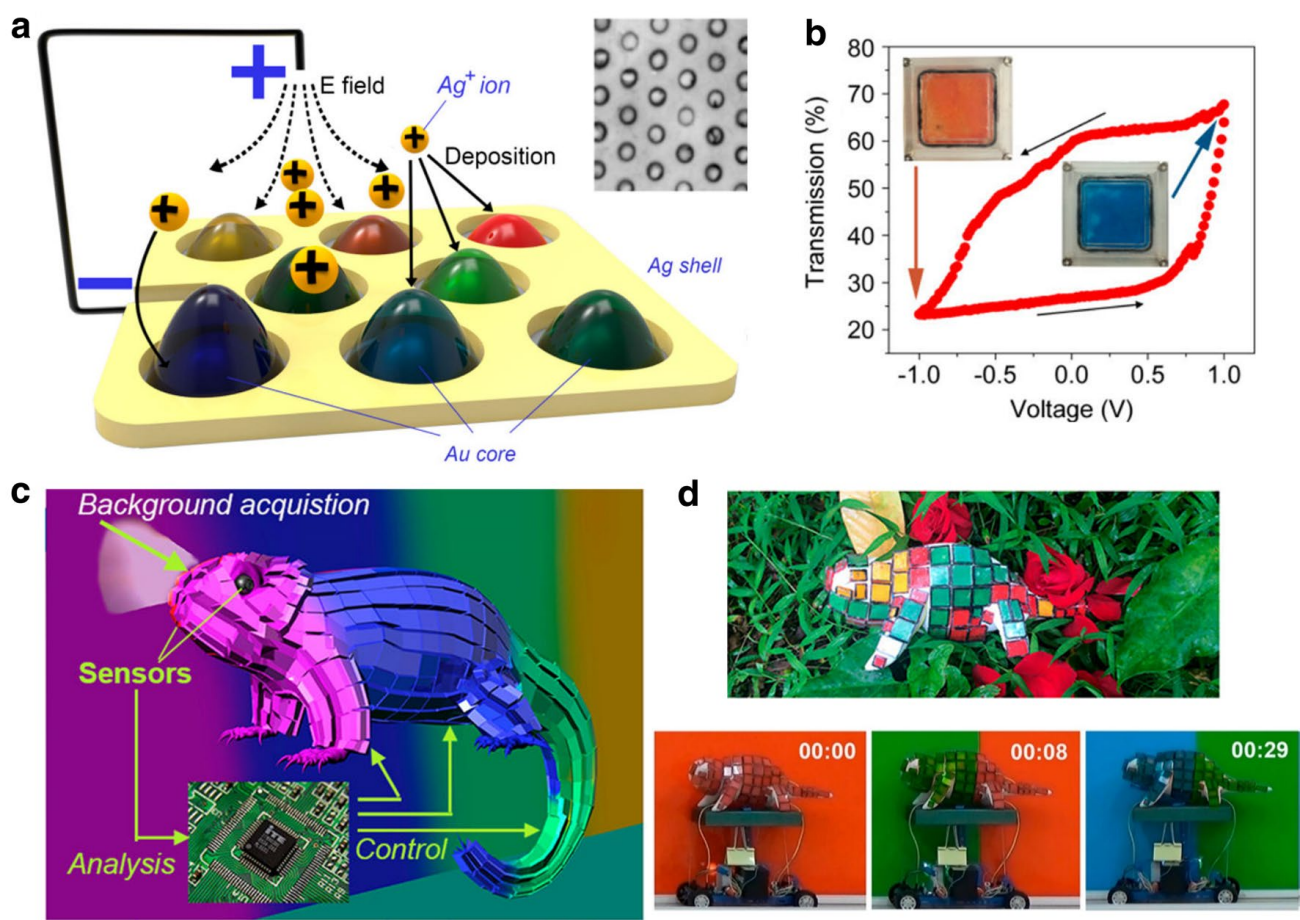
**a** Schematic diagram of plasmonic cell. Inset: SEM image of the Au nanoshell array. **b** Transmission change of unit cell in response to applied voltage. Insets: photos of the cell device points marked by arrows. **c** Schematic of biomimetic camouflage technique obtained using the proposed design. **d** (Top) disguised plasmonic chameleon on outdoor grass. (Bottom) still photographs of camouflage demonstration video [130]

Dynamic plasmonic-nanostructures formed by electrodepositing and electrostripping were evaluated as biomimetic camouflage [130]. The device exploited electrodeposition-induced color transformability of Au/Ag core–shell nanostructures. The plasmonic arrays were composed of a bottom indium tin oxide (ITO) layer, and a perforated SiO_2_ layer with Au/Ag core shells embedded in the holes (Fig. 8a). A plasmonic unit cell is produced by packaging the arrays into a device and filled with gel electrolyte that contain Ag^+^ ions. The coloring process exploits two phenomena: the physical configuration of the plasmonic core shells is tuned by applying a voltage, and the redox reaction of Ag is hysteretic (Fig. 8b). As Ag deposition times increase, the nanodomes inside holes grow, and as a result the cells change color. The plasmonic cells achieved dynamic camouflage (Fig. 8c, d). The plasmonic cells were attached to the body of a mechanical chameleon that was equipped with miniature color sensors. The information from color sensors was analyzed and sent to each cell, and the colors of the chameleon were altered to match the acquired signals. The plasmonic cell device was also fabricated on a flexible substrate; this success shows the possibility of wearable dynamic camouflage.

**Fig. 9:**
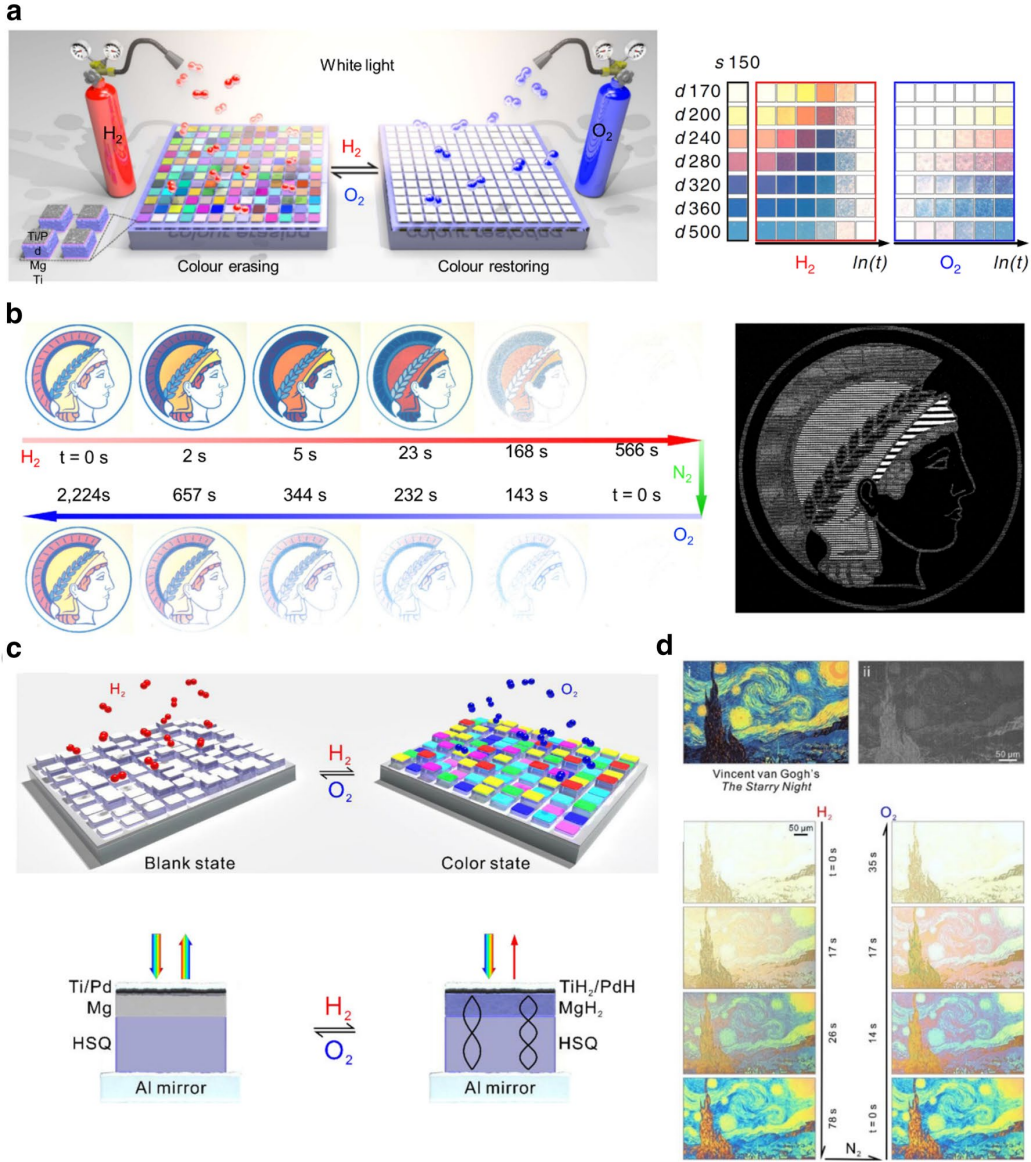
**a** (Left) schematic diagram of plasmonic cell consisting of Mg NPs. White unpolarized light incident on the plasmonic cell in normal direction. H_2_ exposure erases the color; O_2_ restores it. (Right) color palettes as a function of *d* and ln(*t*). **b** (Left) Time-varying color erasing and restoring of fabricated image. (Right) SEM image of the fabricated sample [131]. **c** (Top) Schematic diagram of proposed dynamic stepwise cavity resonators. Upon H_2_ exposure, color information is shown; O_2_ exposure erases the color; H_2_ restores it. (Bottom) cross section view of unit Fabry-Pérot resonator. Under H_2_, a double dielectric spacer is formed and the TiH_2_/PdH layer transmits incident light. **d** (Top) Vincent van Gogh’s Starry Night and fabricated SEM image. (Bottom) color erasing and restoring processes as a function of H_2_ and O_2_ exposure time [132]

Catalytic magnesium (Mg) metasurfaces can be used as dynamic plasmonic color displays [131]. Plasmonic colors were printed, tuned, erased and restored by exploiting time-dependent hydrogenation and dehydrogenation of Mg NPs. When placed between a titanium (Ti) adhesion layer and a Ti/palladium (Pd) complex and exposed to hydrogen, Mg NPs absorb ~ 7.6 wt% hydrogen and is changed from a metal to a dielectric (MgH_2_) (Fig. 9a). The Mg NPs lose their plasmonic features, and reflectance spectra lose all of the features that they showed before the transition. Therefore, hydrogenation can be regarded as an erasing process; dehydrogenation can be regarded as a restoring process. These erasing and restoring processes have possible applications as optical data encryption and decryption (Fig. 9b). This new concept of plasmonic coloring has potential application such as anti-counterfeiting, optical information encryption, and animation printing.

Dynamic displays have been achieved using Fabry–Perot cavity resonators that exploit this metal-to-dielectric transition of Mg [132]. Hydrogen absorption by the Mg layer results in state switch between metal and dielectric; hydrogen desorption causes the reverse process. Upon hydrogenation, a capping layer is changed from Ti/Pd to TiH_2_/PdH, and as a result light can pass through dielectric spacers (MgH_2_ + HSQ) and be reflected by an Al mirror. Then Fabry–Perot resonance modes form in the cavity so that reflected light generates vivid colors (Fig. 9c, bottom, d). Under hydrogen exposure, the color changes, and reaches its final state after 78 s. Under oxygen exposure, the image is restored to the original state within 35 s.

### Mechanical deformation

**Fig. 10:**
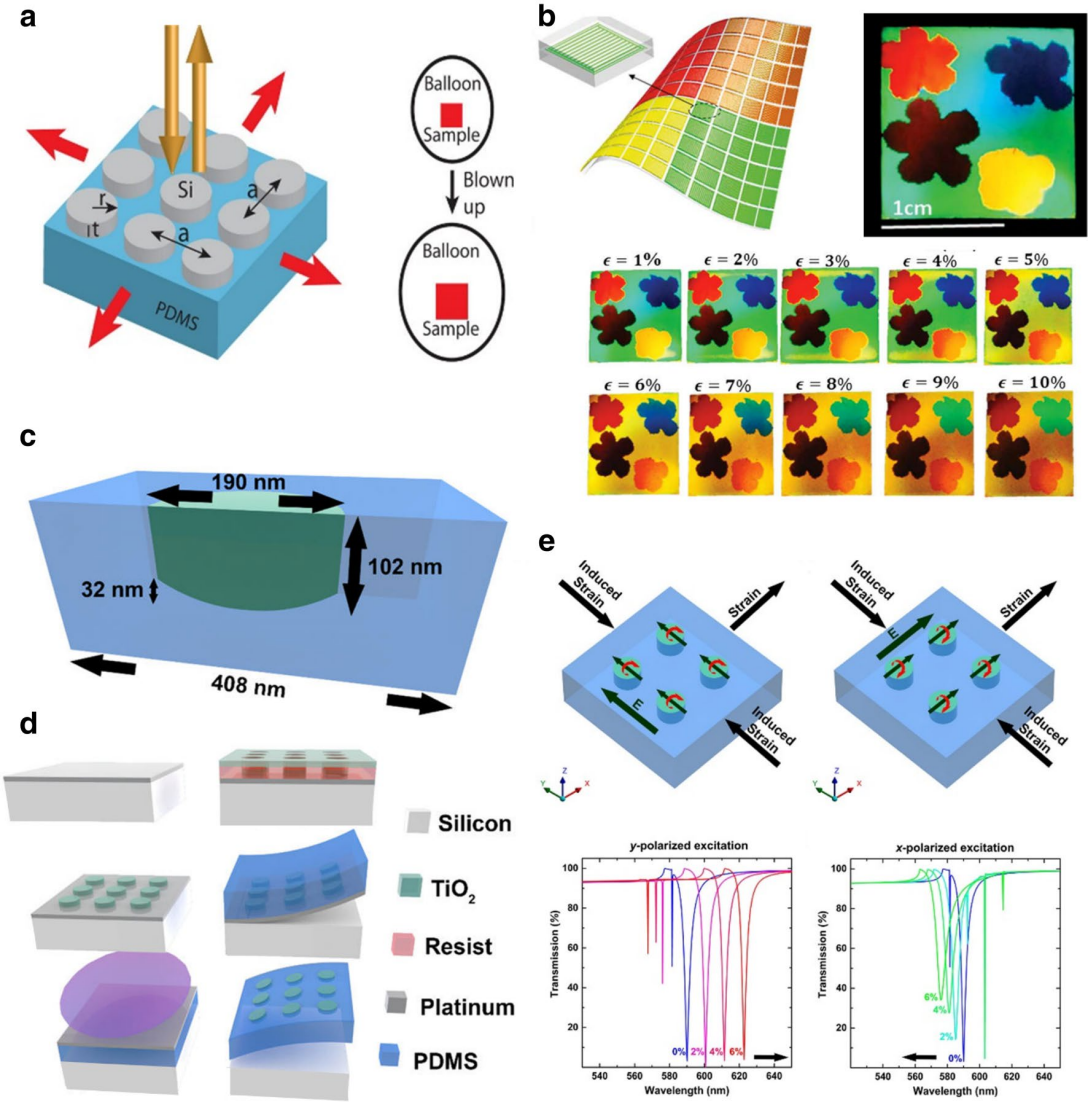
**a** Schematic of mechanically-deformed photonic crystal slap. Inset: Illustration of deformation process [36]. **b** HCM designs and magnified HCM unit cells, and photo of fabricated sample with flower pattern. The scale bar 1 cm. (Bottom) images of color-changing flower as a function of stretching *ε* = Δ*L*∕*L* [37]. **c** Embedded TiO_2_ nanorod in PDMS. **d** Illustration of fabrication process. **e** (Top) *y*-polarized and *x*-polarized dipole orientation with respect to strain direction. (Bottom) *y*-polarized and *x*-polarized transmission spectra with respect to increasing strain. Black arrow: direction of resonance wavelength shifts [38]

Tunability of metasurfaces [39] has applications and possibilities for commercialization. Mechanical deformation is one approach to achieve this tunability. Structural color can be generated by mechanical deformation [36]; specifically by stretching a photonic-crystal slab patterned on a flexible and elastic substrate. This method has achieved resonance-induced reflectance that has sharp resonance peaks; this process is related to Fano resonance [134]. Optical Fano resonance can be regarded as an interference effect between directly reflected light and modes that leak into the environment. Because the reflected light and radiated light have same phase information, constructive interference sharpens the resonance. The reflectance peak can be controlled at will by changing the periodicity *a* in an array of nanodots (Fig. 10a). An array of Si nanorods was patterned on polydimethylsiloxane (PDMS) substrate, then attached to a black balloon to stretch the sample isotropically. Stretching to 10% yielded 32-nm spectral shift; repeated stretching did not cause degradation.

The change in colors can be widened and improved by using a high-contrast metastructure (HCM) composed of metagrating embedded in a transparent and flexible PDMS membrane [37]. HCM pixels were patterned by deep ultraviolet step-lithography, then the Si metagrating was etched to remove the SiO_2_ layer. The HCM was covered by PDMS and detached from SOI Wafer. The resulting PDMS stamp was further protected by encapsulation in a second PDMS layer. Deformation from ε = 0–10%, tuned the color of a flower image fabricated using the suggested structures (Fig. 10b). The HCM has good repeatability under stretching cycles, so this technology may have applications in camouflage and biolabeling.

Mechanical deformation of all-dielectric metasurfaces can also achieve tunable color at visible frequencies [38]. The design was an array of TiO_2_ rods embedded in a PDMS layer (Fig. 10c). The array was patterned by EBL, followed by etching. The PDMS was deposited on top of TiO_2_ array to form embedded TiO_2_ geometry (Fig. 10d). To test color tunability strain was applied the metasurfaces of TiO_2_ resonators in PDMS in two directions orthogonal to each other; with only 6% strain, the resonance peaks shifted 5.08% to red under *x* polarization, and shifted 0.96% to blue under *y* polarization (Fig. 10e, bottom).

## Scalable fabrication for further practical applications

The above-mentioned structural color filters are mainly fabricated by conventional patterning methods such as EBL or focused ion beam milling. These methods seem appropriate for manufacturing subwavelength nanostructures due to an ability to fabricate them elaborately. However, these fabrication methods have limitations such as limited area, low throughput, intricate process, and high cost. In addition, the exceptional potentials and advantages of structural color filters remain a challenge to actualize in practice because of a shortage of scalable and high-speed fabrication methods. If color filtering devices can be fabricated over large area at high throughput, the technologies will have many practical applications and will be useful in major industrial fields [53, 65, 135–139]. Thus, a fabrication method that allows scale-up and fast manufacturing of nanostructures should be developed.

**Fig. 11:**
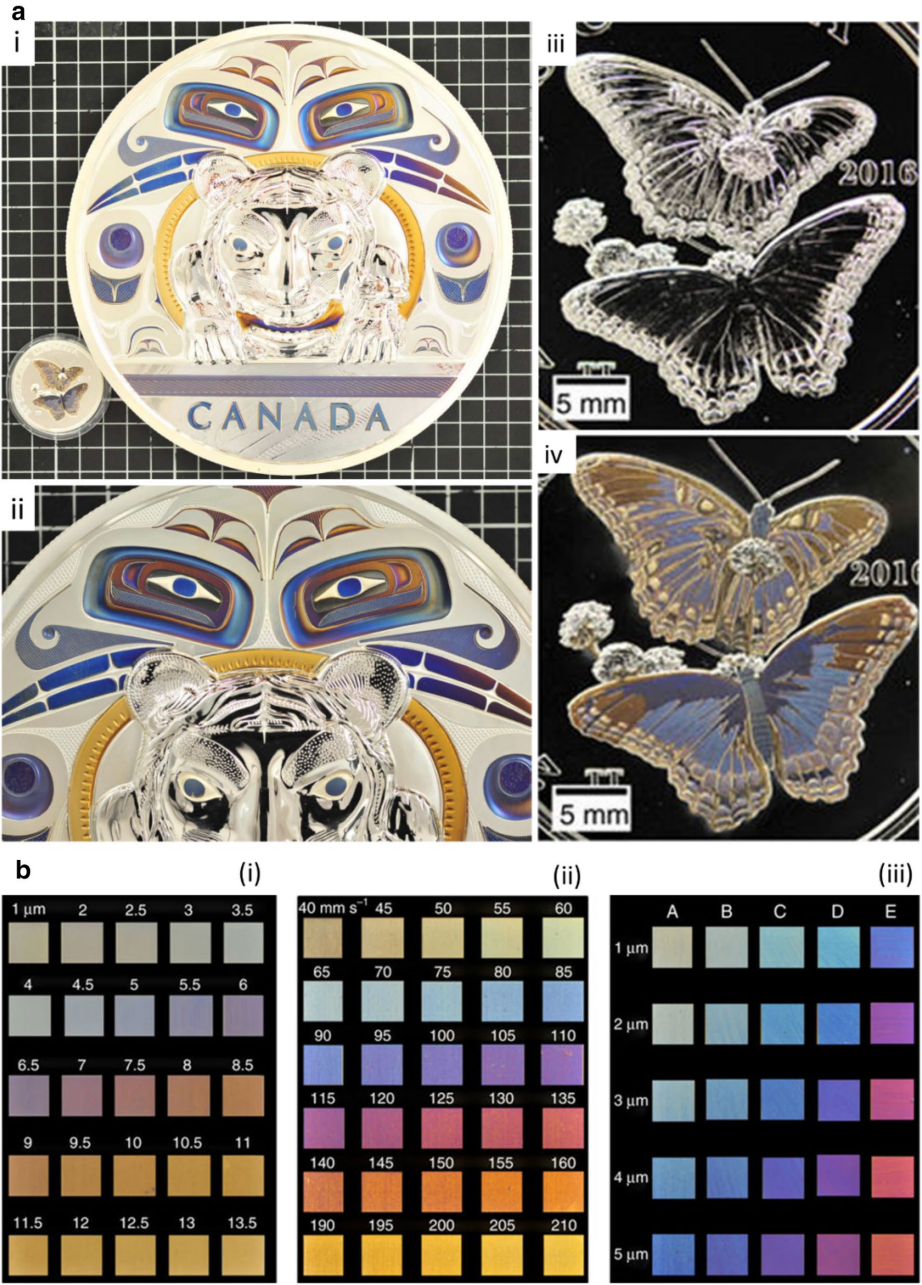
**a** (i) Photograph of a silver-based coin induced by laser with large dimensions of 21-cm diameter and 2.5-cm thickness when compared to butterfly images in detail later. (ii) Close-up image of (i) illustrating detailed coloring. Butterfly shapes on the coin (iii) before and (iv) after laser irradiation. **b** Color palettes at (i) nonburst mode and (ii, iii) burst mode. In (i) 25 colors were produced by varying line spacing in from 1 to 13.5 µm increments of 0.5 µm. In (ii), the color palette was created by tuning machining speed from 40 to 210 mm/s in increments of 5 mm/s. In (iii), the color palette is generated by changing machining angle between machining direction and polarization of light. Column A: 2 passes at 270°; Column B: 1 pass at 270° and 1 pass at 234°; C: 1 pass at 270° and 1 pass at 191°; D: 1 pass at 270° and 1 pass at 162°; E: 1 pass at 270° and 1 pass at 126°. Rows represent spacing between lines 1–5 µm [140]

A laser coloring process with high throughput produces a variety of non-iridescent colors by using ‘normal’ and ‘burst’ modes with a picosecond laser [140]. The picosecond laser is chosen due to lower costs, stronger pulse energies and quicker throughput than existing femtosecond lasers. ‘Normal’ mode uses the laser as usual; in ‘burst’ mode, the same number and energies of pulses are irradiated within a much shorter time than in normal mode to reduce heat damage, increase processing speed, and increase phonon–electron coupling [141–143]. The burst coloring method achieved better quality of colors than the nonburst method (Fig. 11b). Each palette has single a parameter that corresponds to line spacing, laser scanning speed and angle between machining direction and light polarization. The 13.5-µm line spacing and 210-mm/s laser scanning speed at maxima prove that this approach will contribute to practical applications and commercialization of plasmonic color printing.

**Fig. 12:**
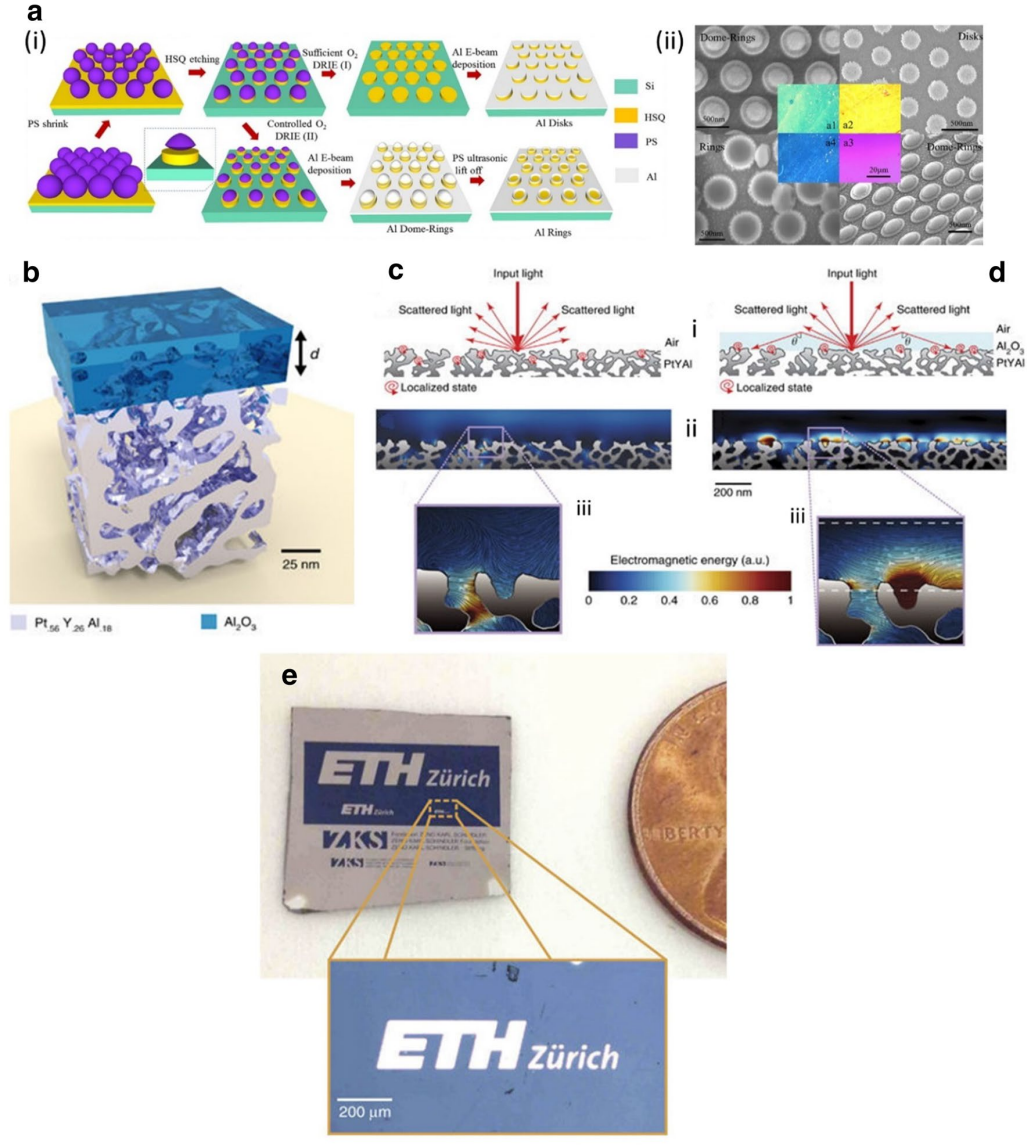
**a** (i) Schematic flows of fabrication steps to produce disk, Al dome-ring and ring structures. (ii) SEM images of structures and corresponding color responses [148]. **b** Schematic design of a network metamaterials based on PtYAl and Al_2_O_3_ layer. **c**, **d** Working principles and calculated energy distributions of network metamaterials without **c** and with **d** Al_2_O_3_ layer. (i) Schematic diagram of interactions between light and matter. (ii) Energy distributions in fabricated structures at *λ* = 425 nm. (iii) Magnified images of (ii) illustrating electromagnetic energy flow indicated by arrows. **e** Photograph of a graphic design based on the network metamaterials. Inset: optical microscopic image showing two uniform colors [149]

Bottom-up methods have widely used to achieve large-area plasmonic colors quickly [144–147]. One such approach exploits colloidal self-assembly fabrication to construct regularly patterned nanostructures with low cost on a centimeter scale [148]. Various structure shapes such as disk, dome-ring and ring can be manufactured according to different fabrication processes (Fig. 12a). To summarize the methods, self-assembled monolayer polystyrene (PS) spheres are transferred onto a spin-coated HSQ layer in accordance with Langmuir–Blodgett approach [145, 146], then the PS spheres are reduced in size by reactive-ion etching. Al disks are obtained by additional O_2_ deep reactive-ion etching (DRIE) and Al e-beam deposition (Fig. 12a, route I). The dome-rings are obtained using short O_2_ DRIE and Al e-beam deposition (Fig. 12, route II). Al rings are created by ultrasonic lift-off to detach the Al dome. The device has many geometric parameters that can be adjusted to tune reflection, such as diameter, height, pitch and width of structures. The device obtained high saturation in the blue region, but low saturation in the green-to-red region. The colors could be adjusted by varying the geometric parameters (Fig. 12a, samples a1–a4). This approach can fabricate 20 samples of 1.5 × 1.5 cm in < 3 h, so it seems suitable for fast mass production.

A novel optical nanomaterial based on large-scale network metasurfaces forms vibrant structural colors varied from thickness of an ultra-thin alumina coating (Fig. 12b) [149]. This approach has biomimetic optical properties inspired by a bird, *Cotinga maynana*, which has blue feathers that are iridescent in a way that cannot be explained by Rayleigh or Mie scattering. A dielectric coating reflects scattered waves to increase scattering, hence generating electromagnetic energy flow and resonant coupling in an Al_2_O_3_ layer (Fig. 12c, d). Color responses and resonant reflectance can be adjusted by modulating the coating thickness, and are blue-shifted as the thickness of the dielectric is increased. The device had high mechanical resistance in a scratch test. A last illustration of figures demonstrates that this approach is compatible with large-area production (Fig. 12e).

## Conclusion and outlook

We have reviewed recent progress in resonance-assisted color generation. Colors achieved using plasmonic resonance and Mie resonance have intriguing features such as exceedingly high resolution, near-permanent lifetime and material simplicity. The resolution may exceed 10^5^ dpi, which surpasses the diffraction limit of light. Resonance-assisted coloring requires only a single or a few nano-size layers, so processing conditions are simple. However, the method has high patterning costs, low throughput and elaborate color tuning mechanisms; these disadvantages must be overcome before commercial applications are possible. Nanoimprinting, self-assembly and laser printing are possible solutions to achieve large-area fabrication and high throughput. LCs, chemical transition and Mechanical deformation may enable accurate and easy color tuning process.

To summarize, resonance-based color printing methods are advancing toward to real-world application. Their features such as ultrahigh resolution, brilliant optical response and compatibility with existing fabrication technologies seem to show a promising future. If fabrication costs can be reduced, and color tuning mechanisms can be improved, these methods will have important potential applications in cryptography, security, imaging, optical data storage and further optical devices.

## Abbreviation

Ag: silver
Al: aluminium
Al_2_O_3_: alumina
Au: gold
a-Si:H: hydrogenated amorphous silicon
c-Si: crystalline silicon
DCFs: dielectric-based color filters
DRIE: deep reactive-ion etching
EBL: electron beam lithography
EOT: extraordinary optical transmission
Ge: germanium
HCM: high-contrast metastructure
HSQ: hydrogen silsesquioxane
ITO: indium tin oxide
LA: long arm
LC: liquid crystal
Mg: magnesium
MIM: metal–insulator–metal
NPs: nanoparticles
PCFs: plasmonic color filters
Pd: palladium
PDMS: polydimethylsiloxane
PS: polystyrene
SA: short arm
SEM: scanning electron microscope
Si: silicon
SP: surface plasmon
SPRs: surface plasmon resonances
SPPs: surface plasmon polaritons
TCFs: tunable color filters
TFT: thin-film transistor
Ti: titanium
TiO_2_: titanium dioxide
